# Amyloid-β and Tau in Alzheimer’s disease: pathogenesis, mechanisms, and interplay

**DOI:** 10.1038/s41419-025-08186-8

**Published:** 2026-01-09

**Authors:** Altaf A. Abdulkhaliq, Bonglee Kim, Yousef M. Almoghrabi, Johra Khan, Amir Ajoolabady, Jun Ren, Suhad Bahijri, Jaakko Tuomilehto, Anwar Borai, Domenico Pratico

**Affiliations:** 1 Department of Biochemistry, Faculty of Medicine, Umm Al-Qura University, Mecca, Saudi Arabia; 2 Department of Pathology, College of Korean Medicine, Kyung Hee University, Hoegidong Dongdaemun-gu, Seoul, Republic of Korea; 3 Department of Clinical Biochemistry, Faculty of Medicine, King Abdulaziz University, Jeddah, Saudi Arabia; 4 Faculty Regenerative Medicine Unit, King Fahd Medical Research Center, King Abdulaziz University, Jeddah, Saudi Arabia; 5 Department of Medical Laboratory Sciences, College of Applied Medical Sciences, Majmaah University, Al Majmaah, Saudi Arabia; 6 National Clinical Research Center for Interventional Medicine, Shanghai, China; 7 State Key Laboratory of Cardiovascular Diseases, Zhongshan Hospital, Fudan University, Shanghai, China; 8 Shanghai Institute of Cardiovascular Diseases, Department of Cardiology, Zhongshan Hospital Fudan University, Shanghai, China; 9 Saudi Diabetes Study Research Group, King Fahd Medical Research Centre, King Abdulaziz University, Jeddah, Saudi Arabia; 10 Food Nutrition and Lifestyle Research Unit, King Fahd Medical Research Centre, King Abdulaziz University, Jeddah, Saudi Arabia; 11 Department of Public Health, University of Helsinki, Helsinki, Finland; 12 King Abdullah International Medical Research Center (KAIMRC), King Saud Bin Abdulaziz University for Health Sciences (KSAU-HS), King Abdulaziz Medical City, Ministry of National Guard Health Affairs, Jeddah, Saudi Arabia; 13 Department of Neural Sciences, Lewis Katz School of Medicine, Temple University, Philadelphia, PA USA

**Keywords:** Alzheimer's disease, Neurodegeneration

## Abstract

Alzheimer’s disease (AD) is a devastating neurodegenerative disease and the most prevalent type of dementia characterized by pathological deposition of amyloid-β plaques/deposits and tau tangles within the brain parenchyma. This progressive ailment is featured by irreversible cognitive impairment and memory loss, often misdiagnosed as the consequence of old age in elderlies. Pathologically, synaptic dysfunction occurs at the early stages and then progresses into neurodegeneration with neuronal cell death in later stages. In this review, we aimed to critically discuss and highlight recent advances in the pathological footprints of amyloid-β and tau in AD. Specifically, we focused our attention on the interplay and synergistic effects of amyloid-β and tau in the pathogenesis of AD. We hope that our paper will provide new insights and perspectives on these pathological features of AD and spark new ideas and directions in AD research and treatment.

## Facts

- Besides aberrant processing of APP (known as Aβ pathology), through activation of largely elusive mechanisms within neurons, hyperphosphorylation of tau protein takes place (involving both known/unknown external and/or internal stimuli), resulting in its dissociation from microtubules and aggregation into tau tangles. These mechanisms should be thoroughly investigated by prospective research.
- The synergistic interplay between Aβ pathology and tauopathy in AD pathogenesis is a well-known phenomenon, suggesting an interconnected or vicious interplay, with speculative and largely unknown mechanisms that could be subject to immense investigation.
- Extracellular oligomers of Aβ and tau act synergistically to damage/alter synaptic function and memory, therefore backing up the hypothesis that Aβ pathology and tauopathy act in parallel through mechanisms other than the amyloid cascade hypothesis, which mainly posits Aβ pathology as the upstream of tau pathology. These notions are encouraging and demand further expansion by future studies.

## Introduction: an overview of the pathophysiology of Alzheimer’s disease

Alzheimer’s disease (AD) is a progressive neurodegenerative disease and the most prevalent form of dementia, impacting memory, behavior, and cognitive abilities [1–3] by distinct neurologically-defined clinical stages as well as the presence of extracellular amyloid beta (Aβ) plaques/deposits and intracellular tau tangles in the brain [4–7]. In the early stage of AD, both Aβ plaques and tau tangles are present, associated with mild cognitive impairment. However, as the disease progresses into the middle and late stages, the cognitive decline becomes more pronounced, featuring the progressive loss of memory, language skills, and behavioral/executive function [8, 9]. Ultimately, the excessive accumulation and growth of Aβ plaques/deposits and tau tangles further exacerbate dementia, cognitive decline, and neuropathological changes, causing neurodegeneration and brain atrophy “(see the Glossary)” [9].

**Fig. 1:**
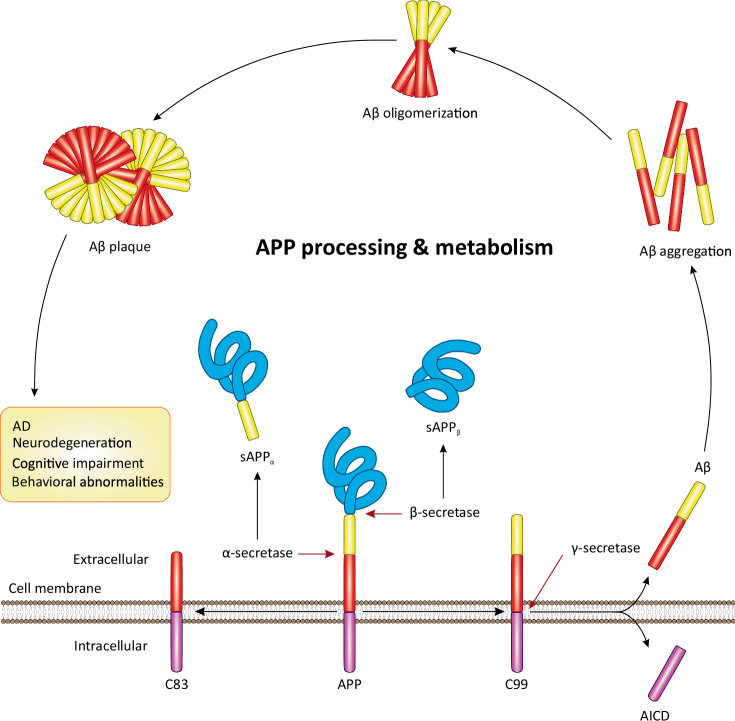
Overview of the processing and metabolism of APP. In neurons, the sequential cleavage of full-length APP by first β-secretase and then γ-secretase generates Aβ peptide as well as sAPP_β_ and AICD components. The accumulative production of Aβ leads to its aggregation and subsequent oligomerization. The oligomerized Aβs then further aggregate, leading to the formation of extracellular Aβ plaques. These plaques drive the pathogenesis of AD and its phenotypes. Alternatively, in the non-amyloidogenic pathway, the cleavage of APP by α-secretase occurs at a site that pertains to the Aβ domain, thereby not yielding an Aβ peptide and instead releasing the sAPP component and leaving the C83 domain embedded in the membrane. Intracellularly, AICD may be further cleaved by caspases, which produce Jcasp and C31 fragments.

Amyloid precursor protein (APP) is a transmembrane protein that is abundantly expressed in the brain, particularly in synapses, where it regulates iron export, neural plasticity, and synapse formation [10]. Under physiological conditions or due to largely unknown pathological stimuli, APP undergoes a series of enzymatic cleavages catalyzed by β-secretase (BACE1) and γ-secretase proteases, which cleave APP from N-terminal and C-terminal sites, respectively, resulting in the production of small Aβ peptides and a soluble secreted fragment known as sAPP_β_ (Fig. 1) [11, 12]. These Aβ peptides exist in two main isoforms: Aβ_40_ (containing 40 amino acids) and Aβ_42_ (containing 42 amino acids) [13, 14]. Notably, Aβ_42_ has two additional C-terminal residues compared to Aβ40, rendering its aggregation [13]. The prevailing hypothesis suggests that the overproduction and aggregation of Aβ, possibly due to the abnormal processing of APP or clearance of Aβ, constitute the primary event in the pathogenesis of AD [11, 12].

**Fig. 2:**
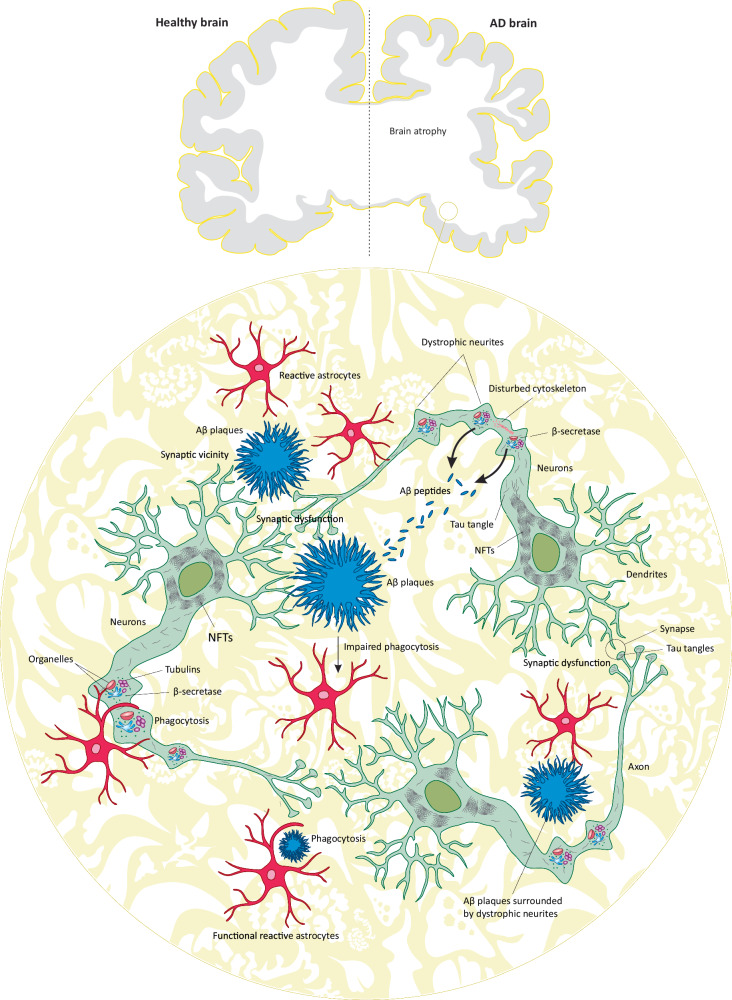
Summary of Aβ and tau pathology in Alzheimer’s disease. AD brain is an atrophic brain characterized by the presence and accumulation of Aβ plaques (mostly extraneuronal) and NFTs within neurons. As shown in the figure, Aβ plaques are usually extraneuronal, surrounded by dystrophic neurites, or may reside in the vicinity of synapses, causing synaptic dysfunction. AD neurons develop dystrophic neurites containing various organelles, accumulated tubulins, and high concentrations of β-secretase. Therefore, rather than other neuronal regions, Aβ peptides are thought to be mainly secreted from dystrophic neurites, which then aggregate as Aβ plaques in interneuronal/extraneuronal space. Functional reactive astrocytes mediate phagocytosis of dystrophic neurites and Aβ plaques to reduce the plaque load and alleviate AD. However, as Aβ plaques continue to rise, they seem to modulate the phagocytic activity of reactive astrocytes through unknown mechanisms, thereby leading to further accumulation of Aβ plaques and exacerbation of AD. On the other hand, tau pathology is an indispensable part of AD pathology. After dissociation from the cytoskeleton, tau proteins form tangles, which then aggregate and propagate throughout neurons, ultimately developing NFTs. In synaptic regions, tau tangles may interfere with axonal transportation.

Aβ peptides are soluble monomers that aggregate as amyloid fibrils, protofibrils, and oligomers during AD pathogenesis [15]. Typically, they form large protease-resistant fibrils, known as Aβ fibrils, which are insoluble and further aggregate to form Aβ plaques (Fig. 2) [15–17]. Additionally, Aβ peptides form diffusible oligomers, known as Aβ oligomers (AβOs), which propagate throughout the brain [15]. AβOs, being more prone to aggregation and propagation, are considered the primary neurotoxic species of Aβ during AD development [15, 18, 19]. The progressive accumulation and deposition of Aβ fibrils and/or AβOs are recognized as one of the pathological hallmarks of AD and one of the main drivers of its pathogenesis [19, 20].

While Aβ peptides may accumulate within neurons and form intraneuronal Aβ aggregates, they are predominantly released into the extraneuronal space (neuronal extracellular matrix) and clump into Aβ fibrils or AβOs, which ultimately form Aβ plaques between neurons (interneuronal Aβ plaques/deposits) (Fig. 2) [21]. It is proposed that in the early stage of AD, the elevated levels of interneuronal Aβ plaques, particularly in the vicinity of synapses, disrupt neuronal/synaptic communication and function [22]. In comparison, the intraneuronal accumulation of Aβ (mainly as AβOs) contributes to neurodegeneration via diverse mechanisms, including the induction of endoplasmic reticulum (ER) stress [23]. However, many of these mechanisms remain poorly understood.

On the contrary, α-secretases are a group of proteases that cleave APP within its transmembrane domain, which contains Aβ. As a result, the processing of APP by β- and γ-secretase proteases does not produce Aβ peptides [24]. Therefore, α-secretases are neuroprotective, and their mechanism of APP processing is often referred to as a non-amyloidogenic pathway. ADAM10 is the primary α-secretase, and its mediated cleavage of APP produces a secreted fragment, known as sAPP_α_, which has received considerable attention in recent years (Fig. 1) [25]. A recently published review has extensively discussed the non-amyloidogenic processing of APP and its neuroprotective role in AD [26].

**Fig. 3:**
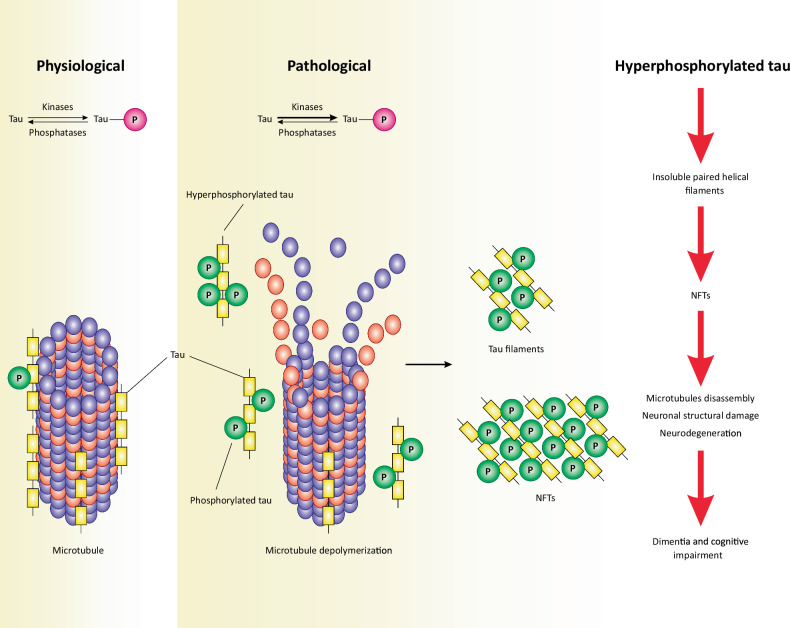
Phosphorylation level of tau regulates the integrity of the neuronal cytoskeleton. Under physiological conditions, there is a balance between the activity of kinases and phosphatases that regulates the phosphorylation level of tau. Due to this balanced activity, tau remains attached to the cytoskeleton and then promotes the assembly and formation of microtubules. This bolsters the structural integrity of the neuronal cytoskeleton. On the contrary, under pathological conditions, kinase reactions are more favored (perhaps due to the increased activity or protein levels of kinases), leading to the hyperphosphorylation of tau. Hyperphosphorylated tau detaches from the cytoskeleton, causing the depolymerization of microtubules, thus impairing the neuronal cytoskeleton. Ultimately, hyperphosphorylated tau proteins self-assemble as tau filaments (paired helical filaments or straight filaments); then, they spread throughout the brain, forming NFTs. These NFTs cause neuronal structural damage or induce neurodegeneration, leading to dementia and cognitive damage.

On the other hand, tau is a microtubule-associated protein predominantly expressed in neurons and primarily localized within axons [27, 28]. Physiologically, tau plays a crucial role in stabilizing the cytoskeleton by promoting the formation of microtubules from tubulin dimers, thereby maintaining neuronal morphology, function, and axonal transport [29–34]. Tau enhances the stability and integrity of microtubules, which sustains the structural integrity of neurons and the intraneuronal transportation of proteins that rely on microtubules as railway tracks (Fig. 3) [29, 34].

However, pathological tau leads to its dissociation from microtubules and subsequent aggregation into tau filaments, which intertwine to form insoluble tau tangles within axons [35, 36]. Additionally, dysfunctional tau impairs the assembly and stability of microtubules, causing architectural damage to neurons and the impairment of intraneuronal cargo transportation by motor proteins, sailing on microtubules [37]. Ultimately, tau tangles propagate throughout the neuronal cell body, culminating in the formation of neurofibrillary tangles (NFTs) (Fig. 2). NFTs obstruct the axonal transportation of nutrients and chemicals, thereby impairing synaptic communications in the central nervous system (CNS) [35, 36]. Synaptic dysfunction likely represents one of the initial events in the pathogenesis of AD [38]. However, the precise mechanisms by which tau leads to neuronal cell death and neurodegeneration remain unexplored in the current literature. Nevertheless, mounting evidence suggests that tau/NFTs play a more significant role in the pathogenesis of AD than Aβ plaques [20, 39, 40].

The general consensus is that the hyperphosphorylation of tau is the primary event rendering the dysfunction of tau in neurons, and the hyperphosphorylated form of tau is one of the pathological hallmarks of AD [41]. The degree of phosphorylation is determined by the activity of kinases and phosphatases involved in tau metabolism. Physiologically, there is an equilibrium between the activity of these enzymes, while pathologically, this balance shifts in favor of kinases, which trigger the hyperphosphorylation of tau (Fig. 3) [41]. Hence, inducing the dephosphorylation of tau or reducing its phosphorylation may restore the phosphorylation balance and homeostasis of tau, which handicaps the development of tau neurotoxicity in AD.

Taken together, Aβ plaques and tau tangles jointly contribute to the initiation and progression of AD. However, their pathological interplay and synergistic effects in AD remain elusive. To this end, here, we aimed to critically discuss the recent seminal findings on the role of Aβ and tau pathology in AD as well as their possible synergism and pathological interplay.

## A closer look at AβOs in AD

### Besides the brain, Aβ oligomerization may occur in the blood

AβOs are the most neurotoxic species of Aβ and thus are deemed more dominant players than Aβ fibrils in the pathogenesis of AD [18, 42, 43]. In healthy individuals and AD patients, as well as patients with cognitive decline and mild cognitive damage, the circulating levels of AβOs were measured to evaluate their impact on brain volume and its structural changes [44]. The results showed that the circulating levels of AβOs were significantly correlated with the reduction of brain volume in different regions/lobes, such as the white matter. However, no causal relationship was discerned [44]. Therefore, Aβ oligomerization not only occurs in the brain within the extraneuronal space but may also occur in the blood, leading to reduced brain volume and the development of AD through unidentified mechanisms. We postulate that in one plausible mechanism, circulating AβOs may trespass into the brain and contribute to the formation of Aβ plaques [45], which induce neurodegeneration and therefore brain loss.

### AβOs and formation of dystrophic neurites

Dystrophic neurites are swollen presynaptic axonal processes with microscopic sprouting stuffed with various organelles/enzymes such as BACE-1 (Fig. 2) [46]. Although several hypotheses have been formulated, mechanisms governing the formation of these structures are largely unknown [47]. Aβ plaques/deposits are in the first line of suspicion for being involved in the formation of dystrophic neurites [47]. This, perhaps, is due to the fact that Aβ plaques are surrounded by dystrophic neurites in AD brains. The in vitro exposure of mouse primary neurons to AβOs (Aβ_42_) was accompanied by the formation of dystrophic neurites, which resembled beads-on-a-string, containing disturbed/immobile microtubules and tubulin aggregates (Fig. 2) [46]. In both AD patients and 5XFAD mice, dystrophic neurites contained disrupted microtubules, mislocalized microtubule proteins (e.g., dynein and kinesin), and high levels of BACE-1 enzyme and Aβ peptides [46]. Besides, autophagy and lysosomes were both defective in dystrophic neurites [46]. Therefore, dystrophic neurites are characterized by the disruption of the cytoskeleton, which blocks axonal transportation and impairs synaptic function. Hence, dystrophic neurites are functional and important components in AD pathogenesis.

Overall, AβOs are probably the main culprit for driving the formation of dystrophic neurites. This notion is supported by the increased presence of BACE-1 in dystrophic neurites, which favors Aβ production, and the dysfunction of autophagy or lysosomes, which prevents the clearance of Aβ. This provides a favorable condition for the formation of AβOs that may precede and drive the formation of dystrophic neurites. However, further studies are warranted to examine the validity of this notion and explore molecular mechanisms, if any, linking AβOs to dystrophic neurites and the exacerbation of AD neuropathology.

### Impaired phagocytosis of AβOs and dystrophic neurites

Reactive astrocytes are a functionally and morphologically modified form of glial cells in the CNS, activated in response to pathological stimuli such as Aβ [48, 49]. Functionally, they encircle Aβ plaques to mediate the phagocytosis/degradation of AβOs as well as dystrophic neurites, which reduces the burden of Aβ plaques in the brain (Fig. 2) [50, 51]. In the hippocampus of APP/PS1 mice containing both dystrophic neurites and AβOs, the phagocytic capacity of reactive astrocytes was significantly declined, thereby both AβOs and dystrophic neurites were preserved [49]. Mechanistically, Aβ peptides were mainly found to reduce the phagocytic capacity of reactive astrocytes through the downregulation of phagocytic receptors (e.g., *MEGF10*/Megf10 and *MERTK*/MERTK) on these cells [49].

In summary, AβOs or peptides reduce the phagocytic activity of reactive astrocytes, thereby preventing their removal and degradation. As a result, the level of AβOs or peptides increases, which further damages the phagocytic capacity of astrocytes, resulting in the amplification of AβOs in a vicious cycle [49, 52]. This notion deserves further investigation to elaborate on the associated molecular mechanisms.

### Synaptic binding, resilience, or sensitization to AβOs

Non-demented with AD neuropathology (NDAN) is a distinct clinical feature of AD characterized by the preservation of cognitive function in patients despite the presence of Aβ species and tau pathology [53, 54]. Hence, studying the preservation of cognitive function in these patients is an interesting research domain that may provide novel therapeutic insights into AD. In a series of studies, the proteomics analysis of hippocampal tissues from NDAN humans revealed a unique post-synaptic proteome compared with that of AD and normal individuals [55, 56]. Moreover, a bioinformatic approach identified 3 upstream regulators, including miRNA-149, miRNA-485, and miRNA-4723, which accounted for the unique proteome observed in NDAN post-synapses. As the gene expression analysis suggested, these miRNAs were differentially expressed in NDAN post-synapses compared with AD and normal post-synapses [55]. Additionally, the in vivo administration of these miRNAs to mice modulated synaptic genes, leading to the reduced binding of Aβ to synaptic terminals and the alleviation of AβOs neurotoxicity [55]. Indeed, these synaptic genes regulated the binding, resilience, or sensitization of post-synapses to AβOs [55]. Overall, it appears that NDAN post-synaptic neurons have a unique neurobiological feature and miRNA profile, which confer resistance against the neurotoxicity and synaptic binding of AβOs. Hence, expanding our knowledge of NDAN post-synaptic neurons should help to reveal other unique features that could be exploited for therapeutic interventions against AD.

Recently, in the temporal cortex of AD patients, synaptic terminals were visualized using a novel microscopy technique [57]. It was revealed that AβOs had potential binding receptors on post-synaptic membranes, including TMEM97, PrPc, and PSD-95, which belonged to a huge protein complex, postsynaptic density (PSD) [57]. Further investigation on APP/PS1 mice and in vitro neurons derived from human pluripotent stem cells revealed that the binding and interaction of AβOs with TMEM97 receptors on post-synapses induced the transcriptional modulation of specific genes, leading to neurodegeneration and neuroinflammation [57]. Hence, TMEM97 is deemed essential for the post-synaptic binding of AβOs and associated neurotoxicity in AD patients [57, 58]. Given that the PSD protein complex contains over 1000 components, such as signaling molecules [59, 60], we postulate that the binding of AβOs to TMEM97 receptor triggers specific signaling pathways in post-synapses, leading to the modulation of gene transcription, thereby driving neurodegeneration and neuroinflammation. Further studies are warranted to reveal these signaling pathways and their mechanism of action.

## The emergence of Tau pathology in AD

### Post-translational phosphorylation of tau

Phosphorylation is the primary post-translational modification of tau protein in the brain under both physiological and/or pathological conditions [61]. Physiologically, the phosphorylation of tau at several serine/threonine residues regulates its binding or dissociation from microtubules [34, 62]. Tau phosphorylation regulates/maintains the stability of the cytoskeleton and axonal transportation in neurons [34]. However, multiple pathological factors (not fully understood) may render tau hyperphosphorylation and dissociation from microtubules, leading to its aggregation and the formation of tau filaments and NFTs (Fig. 3), particularly in axons [63]. Indeed, the hyperphosphorylation of tau restrains its physiological function, causing tau neurotoxicity [31, 64]. Early post-mortem studies on AD brains revealed that tau phosphorylation was elevated by three to fourfold compared with healthy brains [65]. Therefore, the hyperphosphorylation of tau is probably an early event of tau pathology in AD. As such, tau kinases and their implication in tau hyperphosphorylation and mechanisms of activation in neurons are fundamentally important in the discussion of tau pathology in AD.

### PKACb and phosphorylation of tau

*PRKACB*/PKACb is one of the cardinal serine/threonine tau kinases in the brain, contributing to tau hyperphosphorylation and pathology in AD [66]. Based on findings from AD mice (APP/PS1, SAMP8), in vitro cultures, and blood samples from AD patients, the expression of *miR-200a-3p* was downregulated [67]. During AD, *miR-200a-3p* prevented neuronal apoptosis by inactivating the Bax/caspase-3 axis [67]. Additionally, *miR-200a-3p* repressed *BACE1* and *PRKACB* mRNAs, leading to reduced Aβ_42_ content and tau phosphorylation, respectively [67]. Hence, *miR-200a-3p* elicits neuroprotection during AD by inhibiting *PRKACB*/PKACb kinase and tau phosphorylation. However, during AD pathogenesis, *miR-200a-3p* is downregulated, leading to the activation of PKACb tau phosphorylation (or hyperphosphorylation under the prolonged activation of PKACb). Nonetheless, it has not been explored which upstream factors or signaling molecules contribute to *miR-200a-3p* downregulation. Indeed, our current knowledge of PKACb regulation and tau hyperphosphorylation is rather limited; hence, further studies are required in this domain.

### Excitotoxicity and fyn-mediated phosphorylation of tau

*FYN*/Fyn is an intracellular tyrosine tau kinase from the Src family of kinases that plays multiple roles in the CNS [68]. Fyn contains a domain SRC homology 3 (SH3) that binds to Pro-xx-Pro motifs in tau domains [69]. Independent of the interaction with tau, fyn phosphorylates tau at Tyr 18 residues to form Tyr 18-phosphorylated tau in neurons [70]. Meanwhile, the excessive exposure of neurons to glutamate neurotransmitter leads to the hyperactivation of NMDA receptors on neuronal membranes, causing neuronal damage or death [70]. This process is termed “excitotoxicity”, which has been implicated in AD pathogenesis [70, 71]. In mouse hippocampal neurons, Tyr 18-phosphorylated tau enhanced the excitotoxicity by increasing NMDA receptor-mediated Ca^2+^ influx in vitro [70]. Thus, fyn-mediated tau phosphorylation amplifies the excitotoxicity and triggers neurodegeneration. Hence, fyn-induced tau phosphorylation represents a new paradigm underlying tau pathology in AD. Indeed, while tau hyperphosphorylation typically leads to the formation of NFTs, fyn-mediated tau phosphorylation rather promotes excitotoxicity and neurodegeneration. Despite these valuable findings, the current literature has not specifically explored the molecular links between fyn-mediated tau phosphorylation and the pathogenesis of AD. Therefore, immense efforts should be dedicated to this interesting domain of AD research.

### Microglia and GSK3β-mediated phosphorylation of tau

Microglia are highly specialized macrophages of the CNS patrolling the cerebral microenvironment to govern the development, homeostasis, and immunity of the CNS [72]. Under diverse pathological conditions, microglia protect neurons by various means such as phagocytosis, neurogenesis, and the release of anti-inflammatory cytokines [72]. Microglia highly express a transmembrane receptor TREM2, which regulates their function. TREM2 contains an ectodomain that is cleaved by a proteolytic process known as “ectodomain shedding”, which is mediated by sheddase protease. Subsequently, the cleaved ectodomain is released from microglia in a soluble form (sTREM2) [73–75]. In AD patients, sTREM2 levels are increased in the cerebrospinal fluid [75]. A recent study revealed that in cultured mouse neurons, the binding of sTREM2 to transgelin-2 on neuronal membranes induced the phosphorylation of RhoA at Ser 188 residue, thereby inactivating the RhoA-ROCK axis [75]. This inhibited GSK3β, thus preventing tau phosphorylation. As expected, in tau P301S transgenic mice, inducing *TREM2* overexpression or administering sTREM2 inhibited tau phosphorylation and thereby cognitive/behavioral deficits [75]. Therefore, GSK3β is another culprit enzyme involved in tau phosphorylation and AD pathogenesis [3]. The production of sTREM2 and the activation of the RhoA-ROCK-GSK3β axis in neurons serve as a neuroprotective mechanism mediated by microglia to avoid tau hyperphosphorylation as well as AD development. Hence, microglia and their elicited neuroprotective mechanisms against AD tau pathology could be an appealing subject for further research.

### Excessive salt intake and CDK5-mediated phosphorylation of tau

In C56Bl/6 mice, a high-salt diet (HSD) sustainably promoted the phosphorylation and aggregation of tau in the hippocampus and the neocortex, leading to memory deficit and cognitive dysfunction [76]. Mechanistically, HSD increased the activity of cysteine protease calpain-2, which cleaves and activates p35 protein. In turn, p35 binds to and activates CDK5, which phosphorylates tau, leading to its aggregation and, ultimately, cognitive deficit [77]. As such, the administration of CDK5 peptide inhibitor reduced tau phosphorylation, thereby protecting against cognitive deficit [77]. Additionally, calpain’s reduced activity was ascribed to its reduced nitrosylation due to the lack of nitric oxide (NO) production by cerebral endothelial cells (ECs) [77]. In mice, the genetic ablation of *MAPT*/tau reversed cognitive damage, suggesting that tau phosphorylation is essential for HSD-induced cognitive dysfunction [77]. Taken together, HSD reduces NO production by cerebral ECs, leading to the activation of the calpain-2-CDK5 axis, which induces tau phosphorylation associated with cognitive dysfunction [77, 78]. Hence, CDK5 is envisaged as another culprit enzyme mediating tau phosphorylation in AD pathogenesis [77, 79]. Despite these valuable findings, it remains largely unknown how HSD can modulate NO production in cerebral ECs. Thus, further studies are warranted to explore the underpinning mechanisms of HSD-mediated cerebral endothelial dysfunction and tau pathology in AD.

## Phosphatases and tau dephosphorylation in AD

### PPM1B antagonizes DYRK1A kinase and tau phosphorylation

DYRK1A belongs to the protein kinase family of DYRKs, which can phosphorylate multiple serine/threonine residues in tau, causing its hyperphosphorylation, thereby leading to its aggregation within neurons [80]. *DYRK1A* gene is located on chromosome 21 and is overexpressed in Down syndrome due to an additional copy of this chromosome [81]. Hence, DYRK1A-mediated (hyper)phosphorylation of tau is more accentuated in patients with Down syndrome, thus putting them at a higher risk of developing tau pathology and AD [82].

PPM1B also belongs to the protein phosphatase family of PPMs, which can dephosphorylate serine/threonine residues in target proteins [83]. In mammalian cells, using LC-MS/MS and immunoprecipitation techniques, DYRK1A was found to interact with PPM1B [84]. In vitro assays also revealed that for catalytic/kinase activity, DYRK1A autophosphorylates its Ser 258 residue; however, PPM1B dephosphorylates this residue to suppress DYRK1A activity, thus rendering its inability to phosphorylate tau at Thr 212 residue [84]. This prevented the oligomerization, aggregation, and toxicity of tau in various cell lines, including *DYRK1A*-overexpressing H19-7 cells (hippocampal progenitor cells, widely used in AD studies) [84]. Nonetheless, tau is not a specific substrate for DYRK1A. Besides, except for Down syndrome patients, *DYRK1A* overexpression may not occur in other AD patients. Hence, DYRK1A may not be a predominant tau kinase during AD pathogenesis. Nonetheless, this study successfully revealed that PPM1B phosphatase negatively regulates DYRK1A tau kinase in vitro, thereby protecting against tau pathology and AD. Hence, the study on phosphatases that regulate tau kinases during AD pathogenesis is an encouraging research domain, but still in its infancy.

### PGA1 induces PP2A-mediated dephosphorylation of tau

PP2A is a predominant phosphatase in the human brain that can dephosphorylate tau at various serine/threonine residues under physiological conditions; however, its activity decreases by 50% in AD brains [85–87]. This reduction is associated with the hyperphosphorylation of tau, neurodegeneration, and memory deficits in rat brains [88].

PGA1 is also a neuroprotective metabolite derived from the plasma membrane phospholipids through chain reactions catalyzed by PLA2 and COX-2 enzymes [89–91]. PGA1 elicits neuroprotection by upregulating the gene expression of neurotrophic factors and regulating PP2A activity [92, 93]. In tau P301S transgenic mice, the intracerebroventricular administration of PGA1 prevented tau phosphorylation at different serine/threonine residues in a dose-dependent manner [93]. In tau mice and in vitro Neuro2a cells, PGA1-mediated activation of PP2A led to tau dephosphorylation [93]. PP2A protein complex contains three subunits, including subunit A (scaffold), B (regulatory), and C (catalytic), among which subunits A and C are primarily required for the activation of PP2A and tau dephosphorylation [94]. Mechanistically, PGA1 formed a covalent bond with Cys 377 residue in subunit A via a chemical reaction known as the “Michael adduct reaction” [93]. The long-term administration of PGA1 to tau P301S transgenic mice (6 months, 2.5 mg/kg/day) averted cognitive decline [93]. Taken together, PP2A is a key tau phosphatase that prevents tau pathology in AD. Besides, PGA1 positively regulates PP2A activation; thus, its elevation in neurons may promote tau dephosphorylation and alleviate AD.

## The interplay between amyloid-beta and Tau pathology in AD

### Evidence for the synergistic effects of Aβ and tau pathology

Mouse APP/PS1+tau model recapitulates Aβ pathology and the overexpression of human *MAPT*/tau [95]. In a seminal study using APP/PS1+tau mice, both Aβ plaques/deposits and tau pathology were found to contribute to the development of aging-associated hyperactive behavioral phenotype [95]. RNA-sequencing analysis revealed that APP/PS1+tau mice had 1,531 differentially expressed transcripts in comparison with control mice [95]. Given that gene expression modulation was larger in APP/PS1+tau mice in comparison with control, APP/PS1, and tau mice, Aβ plaques and tau pathology were perceived to synergistically modulate neuronal transcriptome [95]. In APP/PS1+tau mice, the majority of upregulated transcripts were expressed in glial cells, involved in neuroinflammation [95]. However, the majority of downregulated transcripts were involved in synaptic function [95]. Also, the upregulated genes were largely but independently driven by Aβ or tau, while the combination of the two exerted synergistic effects on downregulated genes [95]. Interestingly, the upregulated genes in microglia enabled them to phagocytize synapses, which may explain the downregulation of synaptic genes in APP/PS1+tau mice [95]. Nonetheless, lowering tau halted transcriptional changes and ameliorated hyperactive behavioral phenotype in APP/PS1+tau mice [95]. This study (perhaps for the first time) revealed that Aβ plaques and tau pathology synergistically promote the development of AD and its phenotypes [95, 96]. This study drew its conclusions mainly from comparative analyses of transcriptomic profiles between the different genotypes of mice. However, regarding the molecular mechanisms, it remains to be found out if and how Aβ plaques and tau pathology are interlinked or synergistically modulate gene transcription in the glia or neuronal synapses. Besides, we believe that the model used in this study (APP/PS1+tau mice) only entails the overexpression of *MAPT*/tau, which may not necessarily recapitulate the phosphorylation or aggregation of tau, and therefore tau pathology. Hence, it seems that the APP/PS1+tau model does not accurately recapitulate tau pathology in AD; thus, it may not be suited for studying the interplay between Aβ and tau pathology in AD.

In another key study, a better mouse model was introduced that could better remodel tau pathology (tau aggregation and the formation of NFTs) along with Aβ plaques in AD [97]. In 6–12-month-old APP/PS1 mice, bearing Aβ plaques without tau pathology, neuronal hyperactivity was increased in layer 2/3 of the neocortex [97]. However, in age-matched transgenic rTg4510 mice displaying tau aggregation and NFTs without Aβ plaques, cortical activity was markedly reduced, and silent neurons were upregulated up to 3.6 to 5.8 fold [97]. Immunohistochemistry staining revealed that only 1.21% of layer 2/3 neurons from the neocortex had NFTs, suggesting the pathological role of soluble tau in silencing neurons [97]. To recapitulate both Aβ and tau pathologies, APP/PS1 mice were crossed with rTg4510 mice, and in the new strain, both neuronal hyperactivity and cortical activity were significantly reduced regardless of mouse age [97]. Therefore, the cortical co-presence of tau and Aβ prevents Aβ-associated hyperactivity, causing the profound silencing of neuronal circuits [97]. This suggests that tau pathology predominates the effects of Aβ plaques in cortical neurons. Also, the downregulation of *MAPT*/tau expression or protein level did not rescue neuronal silencing due to the presence of Aβ [97]. These findings provide other compelling evidence that Aβ and tau pathology confer synergistic effects on the silencing and impairment of neuronal circuits in vivo. Yet, such notions (tau pathology predominating the effects of Aβ plaques or the synergistic effects of Aβ plaques and tau on neuronal silencing) require further studies with a specific focus on the underlying molecular mechanisms [96, 98]. Technically, this study ([97]) provides a general insight into these notions but poorly delves into the underpinning molecular mechanisms.

### Evidence for the involvement of Aβ in promoting tau pathology

In an early study, the administration of Aβ_42_ into the brains of P301L mice (transgenic tau mice) elevated NFTs in neuronal cell bodies by 5-fold [99]. Additionally, tau proteins in NFTs were phosphorylated at Ser 212, Thr 214, and Ser 422 residues [99]. This study did not provide a mechanistic insight into the pathological interplay between Aβ and tau in the context of AD; however, for the first time, it revealed that Aβ peptides are seemingly engaged in promoting tau phosphorylation and the formation of NFTs in AD.

In an independent study, tau paired helical filaments (PHFs), also known as tau fibrillar aggregates, were extracted from the human postmortem AD brains; then, administered into the cortices of wild-type and 5xFAD mice (a transgenic model of Aβ plaques) [100]. This induced a working memory deficit in 5xFAD mice [100]. Importantly, PHFs provoked the phosphorylation, oligomerization, aggregation, and propagation of endogenous tau (the 4 R isoform) in the cortical dystrophic neurites of 5xFAD mice in comparison with wild-type mice [100]. In addition, the cortical activity of CDK5 (a potential enzyme involved in tau phosphorylation) was comparably higher in 5xFAD than in wild-type mice [100]. Given that the size and accumulation of oligomeric tau were significantly greater in dystrophic neurites in the vicinity of Aβ plaques than in distant neurites, it was projected that Aβ plaques were likely involved in PHFs-mediated oligomerization and accumulation of endogenous tau in 5xFAD mice [100]. Similar to the previous work, this relatively recent study did not explore the molecular mechanisms underlying PHFs- and Aβ-mediated induction of tau pathology (tau phosphorylation, aggregation, and propagation) in the cortices of 5xFAD mice. However, these findings provide compelling evidence for the implication of Aβ plaques in tau pathology upon the presence or following the administration of PHFs. Furthermore, an intriguing concept inferred from this study is that tau proteins or aggregates propagate between synaptically connected neurons via largely unknown mechanisms [100–102]. Here, it appears that exogenous tau was the first provocation for driving tau propagation and the exacerbation of tau pathology in the mouse cortices [100]. Hopefully, future research may elaborate on these notions, particularly from the perspective of molecular mechanisms.

### Aβ and tau interactions: a concept on the horizon

In an observational study, neuroimaging techniques were employed to monitor tau-positive cortical regions in the human brain [103]. In presymptomatic AD individuals, displaying normal cognitive function, the entorhinal cortex was found to be the early site for the formation of NFTs and tau pathology [103, 104]. Prodromal AD patients, who exhibit mild cognitive dysfunction, were also selected to monitor tau pathology and its progression. The results showed that the propagation (nonlinear spreading) of tau was accelerated in the prodromal stage of AD [103]. Using the network flow-based model, it was shown that the inferior temporal gyrus in the cortical temporal lobe was the primary hub for tau propagation and spreading [103]. Then, the authors attempted to explore potential interactions between Aβ and tau in the cortices. As a result, two interaction modes were proposed, including the remote and local [103]. In the remote interaction, Aβ-positive neurons synaptically interacted with tau-positive neurons, causing neurotoxicity on both sides. The remote interaction was shown to occur in the lateral entorhinal cortex, thereby instigating and promoting tau propagation [103]. In the local interaction, Aβ and tau directly interacted and co-mingled, mainly in the left and right inferior temporal gyrus. The local interaction was proposed to account for the escalation of tau propagation [103]. However, this key study was basically an observational neuroscience work and revealed the interaction pattern between Aβ and tau. In summary, in the lateral entorhinal cortex, the remote interaction between Aβ and tau triggers tau pathology. When the remote interaction switches to the local interaction in the inferior temporal gyrus, tau pathology progresses, as shown by increased tau propagation. Despite these valuable findings, we are still far behind in fully understanding how Aβ-tau interaction in either mode can promote tau pathology or cause its propagation. Hence, further studies are warranted to fill in the gaps and provide elaborated mechanistic insights into events taking place in the brain cortices in presymptomatic or prodromal AD patients.

The accumulating number of evidence suggests that tau proteins are released from neurons mainly through synaptic exosomes [105–107]. In a key study, the frontal and parietal cortex samples obtained from human postmortem AD patients were utilized to prepare cortical synaptosomes. These synaptosomes were depolarized then the degree of depolarization was quantified [108]. Indeed, the depolarization of cortical synaptosomes was a technique to prompt the release of extracellular vesicles such as exosomes and tau. The synaptosome-released tau was oligomeric, c-terminally truncated, and encapsulated by exosomes [108]. The FRET (fluorescence resonance energy transfer) biosensor assay revealed that exosomal oligomeric tau exhibited seeding/propagation activity. However, free-floating synaptosome-released tau did not exhibit such activity [108]. Moreover, the findings of this study, as well as other works, suggest that besides tau, synaptic exosomes may also contain toxic oligomeric Aβ [108–110]. In this regard, the FRET biosensor assay demonstrated that oligomeric Aβ, particularly, Aβ_42_ was strongly correlated with the seeding/propagation activity of exosomal tau [108]. Therefore, both oligomeric tau and Aβ are perceived to be released from cortical synapses via synaptic exosomes. Upon their release, we postulate that exosomes containing Aβ likely and preferentially deposit in the neuronal extracellular environment, forming Aβ plaques, while exosomes containing tau more likely traverse between pre and postsynaptic neurons to transport tau between them, leading to the cortical spread/propagation of tau.

In this exosomal model, we proposed that both the local and remote interactions may occur between Aβ and tau. In one possible scenario, the remote interaction may occur when exosomes containing Aβ and/or tau traverse between synapses or float in the synaptic gap. Alternatively, the local/direct interaction may occur when exosomes (particularly those containing tau) reach the membrane of a target synaptic neuron and release their content. Supposedly, these interactions favor the interneuronal spreading and propagation of tau, thereby exacerbating AD pathogenesis. Despite adequate evidence for the interaction between Aβ and tau in AD pathogenesis, we are still far behind in comprehensively understanding the molecular mechanisms underpinning such interactions. Hence, AD researchers are urged to switch their focus from observational to more basic studies in this evolving domain of AD research.

## Promises of immunotherapy against Aβ and tau pathology in AD

Currently, we fall short of introducing proper AD treatments that could significantly reverse the disease progression, thus have only sufficed with some short-term remedies that alleviate the symptoms, such as cognitive and memory problems/abnormalities [111]. Moreover, the intricate pathophysiology of AD (which, here in this review, we tried to echo) further complicates the development of impeccable therapies against AD [111, 112]. To this end, immunotherapy is now being gazed upon as one of the promising therapeutic slots against Aβ and tau pathologies in AD [112]. In a simple term, immunotherapies implement monoclonal antibodies (mAbs) or synthetic peptides to lighten the load of Aβ plaques in the brain through provocation of immune cells/responses and subsequent breaking down/clearance of the plaques, thereby halting or slowing down AD progression [112, 113]. In a recent post hoc analysis of a double-blinded, parallel-group, multicenter, and placebo-controlled phase 2 randomized clinical trial, ADAMANT (for 24 months) (EudraCT2015-000630-30, NCT02579252) with 196 AD patients, 119 were selected and included in the analysis [114]. AADvac1 is an active immunotherapy vaccine eliciting an immune response against pathological forms of tau [114, 115]. Moreover, machine learning models were employed to predict/detect both Aβ^+^ and tau^+^ patients from the baseline MRI. The MMRM (a mixed model for repeated measures) statistical method was used for scrutiny of baseline changes in neurodegeneration and cognitive function, and finally, linear regression analysis of the link between endpoints and antibody response [114]. The results showed that the prediction model provided the PPV (positive predictive value) of 96.2% and 97.7% for tau and Aβ, respectively. Also, the plasma levels of NF-L (plasma neurofilament light), a biomarker of axonal damage, were depleted (*p* = 0.0139). Moreover, the greater AADvac1 antibody response was associated with increased CDR-SB (an endpoint for measuring cognitive changes) (*p* = 0.0376) and ADL (activities of daily living) (*p* = 0.0201), an indicator of fundamental skills for independent taking care of oneself, and reduced brain atrophy (*p* < 0.05) [114]. Overall, these findings denote the promises of AADvac1 immunotherapy against tau for alleviation or reversal of tau pathology and symptoms in AD patients; nonetheless, further clinical trials on a larger scale are warranted.

On the other hand, CAD106 is an active immunotherapy vaccine against Aβ [111, 116]. Besides, BACE1 inhibitors such as umibecestat are potential anti-AD drugs antagonizing the production of neurotoxic Aβ [117, 118]. The Alzheimer’s Prevention Initiative (API) Generation Study 1 (GS1) (NCT02565511) was another double-blinded, parallel-group, two-cohort, and placebo-controlled randomized trial, evaluating CAD106 and umibecestat in 60–75-year-old participants (cognitively unimpaired but harboring genetic risk factors of AD), which was terminated early [119, 120]. In this trial, the results from the CAD106 cohort revealed that CAD106 increased serum levels of Aβ immunoglobulin G in 41 of 42 participants, reduced the formation rate of Aβ plaques (*P* < 0.001), and moderated three cases of ARIA (Aβ-related imaging abnormalities) [119]. Therefore, despite the early termination of this trial, their findings still urge the execution of larger trials on active Aβ immunotherapies in those individuals genetically prone to AD development.

## Conclusion and final remarks

On the neuronal membrane, APP proteins undergo abnormal enzymatic cleavage, leading to the exaggerated production of Aβ peptides and their ultimate aggregation into Aβ plaques in the extraneuronal space. Different Aβ species, such as AβOs, interfere with the normal function of neurons by various means, ultimately, causing neurodegeneration. Aβ pathology has long been regarded as the primary driver of AD pathogenesis, a concept that remains true today. However, the flip side of the coin reveals tau pathology. Physiologically, tau proteins assist in maintaining the integrity and function of microtubules. Yet, akin to the aberrant processing of APP, the activation of largely unknown mechanisms within neurons (likely triggered by external and/or internal stimuli) leads to tau hyperphosphorylation, leading to its dissociation from microtubules and aggregation into tau tangles. As a result, tau tangles spread throughout neurons, ultimately forming NFTs. Tau tangles disrupt the synaptic communication between neurons and the cytoskeleton as well as compromise the morphological integrity of neurons, resulting in synaptic/neuronal dysfunction.

Historically, compared with Aβ, tau pathology was considered the secondary factor in AD pathogenesis. However, now, ample evidence corroborates that these two pathologies are equally important and exert synergistic effects on AD pathogenesis. Some evidence also suggests the interconnected or vicious interplay between these two pathologies in mouse models harboring both Aβ and tau pathology. This is a crucial point, and throughout the main text, we aimed to discuss how extracellular oligomers of Aβ and tau act synergistically to impair synaptic function and memory, thereby supporting the hypothesis that these two pathologies act in parallel through mechanisms other than the amyloid cascade hypothesis, which mainly posits Aβ pathology as the upstream of tau pathology. In this review, we highlighted these concepts by critically examining and discussing recent seminal works. Ultimately, we encouraged ourselves and our colleagues to delve into the molecular aspects of these pathologies to enhance the therapeutic translation of such findings and strengthen our fight against AD.
